# Preschool Obesity Is Associated With an Increased Risk of Childhood Fracture: A Longitudinal Cohort Study of 466,997 Children and Up to 11 Years of Follow‐up in Catalonia, Spain

**DOI:** 10.1002/jbmr.3984

**Published:** 2020-04-07

**Authors:** Jennifer CE Lane, Katherine L Butler, Jose Luis Poveda‐Marina, Daniel Martinez‐Laguna, Carlen Reyes, Jeroen de Bont, Muhammad Kassim Javaid, Jennifer Logue, Juliet E Compston, Cyrus Cooper, Talita Duarte‐Salles, Dominic Furniss, Daniel Prieto‐Alhambra

**Affiliations:** ^1^ NIHR BRC, Nuffield Department of Orthopaedics, Rheumatology and Musculoskeletal Sciences (NDORMS), University of Oxford Oxford UK; ^2^ Department of Trauma and Orthopaedic Surgery Stoke Mandeville Hospital Aylesbury UK; ^3^ GREMPAL Research Group, Fundació Institut Universitari per a la Recerca a l'Atenció Primària de Salut Jordi Gol i Gurina (IDIAPJGol) and CIBERFes, Universitat Autonoma de Barcelona and Instituto de Salud Carlos III Barcelona Spain; ^4^ Department of Metabolic Medicine Lancaster Medical School, Lancaster University Lancaster UK; ^5^ Department of Medicine Cambridge Biomedical Campus Cambridge UK; ^6^ MRC Lifecourse Epidemiology Unit University of Southampton Southampton UK

**Keywords:** PEDIATRICS, EPIDEMIOLOGY, FRACTURE PREVENTION, FRACTURE RISK ASSESSMENT, NUTRITION, OBESITY

## Abstract

This study aimed to determine if having an overweight or obese range body mass index (BMI) at time of beginning school is associated with increased fracture incidence in childhood. A dynamic cohort was created from children presenting for routine preschool primary care screening, collected in the Information System for Research in Primary Care (SIDIAP) platform in Catalonia, Spain. Data were collected from 296 primary care centers representing 74% of the regional pediatric population. A total of 466,997 children (48.6% female) with a validated weight and height measurement within routine health care screening at age 4 years (±6 months) between 2006 and 2013 were included, and followed up to the age of 15, migration out of region, death, or until December 31, 2016. BMI was calculated at age 4 years and classified using WHO growth tables, and fractures were identified using previously validated ICD10 codes in electronic primary care records, divided by anatomical location. Actuarial lifetables were used to calculate cumulative incidence. Cox regression was used to investigate the association of BMI category and fracture risk with adjustment for socioeconomic status, age, sex, and nationality. Median follow‐up was 4.90 years (interquartile range [IQR] 2.50 to 7.61). Cumulative incidence of any fracture during childhood was 9.20% (95% confidence interval [CI] 3.79% to 14.61%) for underweight, 10.06% (9.82% to 10.29%) for normal weight, 11.28% (10.22% to 12.35%) for overweight children, and 13.05% (10.69% to 15.41%) for children with obesity. Compared with children of normal range weight, having an overweight and obese range BMI was associated with an excess risk of lower limb fracture (adjusted hazard ratio [HR] = 1.42 [1.26 to 1.59]; 1.74 [1.46 to 2.06], respectively) and upper limb fracture (adjusted HR = 1.10 [1.03 to 1.17]; 1.19 [1.07 to 1.31]). Overall, preschool children with an overweight or obese range BMI had increased incidence of upper and lower limb fractures in childhood compared with contemporaries of normal weight. © 2020 The Authors. *Journal of Bone and Mineral Research* published by American Society for Bone and Mineral Research.

## Introduction

The increasing prevalence of childhood obesity is a major public health concern worldwide.^1^, ^2^ Childhood obesity has previously been associated with cardiorespiratory disease, diabetes, and mental health disorders in later life, in addition to premature mortality.^3^, ^4^, ^5^, ^6^, ^7^ Research to date has largely focused upon the impact of obesity in later childhood and the impact on adult health, with less known about the effect of preschool obesity upon health in childhood and adolescence.

Observational studies in adults have implicated obesity in the pathophysiology and outcome of fractures, with protection against fracture at some anatomical sites and increased risk at others.^8^, ^9^, ^10^ Fractures are very common in childhood, with associations found with male sex, team sport participation, urban living, ethnicity, and socioeconomic deprivation.^11^, ^12^, ^13^, ^14^, ^15^, ^16^ Chronic kidney disease, low bone mineral density, glucocorticoid use, vitamin D deficiency, and dietary factors have been implicated in the pathophysiology of fracture.^17^, ^18^, ^19^, ^20^, ^21^, ^22^ Vitamin D deficiency has been associated with childhood obesity, obesity being implicated in pediatric fracture pathophysiology in retrospective clinical and basic science studies.^23^, ^24^, ^25^, ^26^, ^27^, ^28^, ^29^, ^30^, ^31^

Less evidence exists for the impact of preschool obesity upon future health, with previous studies including a wide age range of children producing conflicting results.^32^ A focused study of the association between preschool obesity and fracture risk offers the opportunity to better understand the impact of obesity in early life. Observational data, especially whole region population data linked to routine clinical care, offer the opportunity to study trends in childhood pathology that ethically may be difficult in a trial setting and to include subgroups of patients who may either be excluded from or underrepresented in clinical trials.

The primary aim of this study was to determine if elevated body mass index (BMI) just before starting school at age 4 years is associated with an increased incidence of fracture in childhood. Secondly, we aimed to analyze the association between BMI and the anatomical site of fracture.

## Materials and Methods

### Study design and setting

A prospective dynamic cohort was made using anonymized primary care electronic health records from the Information System for Research in Primary Care (SIDIAP; www.sidiap.org), including the data from the pediatric health care program.^33^ SIDIAP is based in the region of Catalonia, Spain, where health care is universal, paid for by taxation. A total of 296 primary care centers with 853 primary care pediatricians in Catalonia contribute to SIDIAP, covering more than 74% of the total population. Data contained in SIDIAP has been found to be representative of the Catalan population in previous studies comparing electronic health records with health surveys, and strict criterion are used to ensure data quality is maintained with the data set.^33^, ^34^, ^35^

The pediatric health care program is a comprehensive surveillance of childhood growth and development in all children in the region. All children in the region are regularly reviewed by primary care pediatricians and pediatric nurses from birth until they transition to general practitioner care at age 15 years. Data on weight and height measurements between January 1, 2006, and December 31, 2013, were included in this study.

In 2019, Catalonia represented 16.2% of the overall Spanish population.^36^ Of the Catalan population, 15.5% were estimated to be children aged 0 to 14 years, which is similar to the estimate average in the European Union (EU; 15.6%). It was estimated that there are 104.2 women per 100 men in Catalonia, again similar to the rest of the eurozone (104.5 women per 100 men).^37^ Catalonia is estimated to have 10.9% unemployment, lower than in Spain overall (14.2%) but higher than the average in the EU of 6.3%.

### Population

All children assessed at the school starting age of 4 years (± 6 months) by a pediatrician or pediatric nurse in any of the contributing Catalan Health Institute primary care centers were included. Participants must have had at least one valid height and weight measurement recorded within the recruitment period to be included. Height and weight were used to calculate BMI (kg/m^2^). These measurements were taken as part of routine clinical care within a pediatric health surveillance program within the region.^38^

Participants were followed up from the date of index BMI measurement until they reached age 15 years, migrated out of the SIDIAP region, or died, or until the end of the study period (December 31, 2016).

### Study exposure and outcomes

The main exposure of the study was BMI category (underweight, normal weight, overweight, and obesity) of the children at age 4 years (± 6 months). BMI category was obtained by calculating age‐ and sex‐specific BMI *Z*‐score (number of standard deviations from the reference population) following the World Health Organization (WHO) growth standard.^39^ These growth standards were determined by the WHO Multicenter Growth Reference Study collecting data from healthy child populations in Brazil, Ghana, India, Norway, Oman, and the USA, therefore representing a wide range of ethnic backgrounds.^40^ The categories were defined as: underweight (<−2 BMI *Z*‐score), normal weight (−2 to +2 BMI *Z*‐score), overweight (> +2 *Z*‐score), and obesity (> +3 *Z*‐score). Biologically implausible values of height, weight, and BMI were removed according to the WHO guidance, and a conditional growth percentile model up to age 10 years was applied to remove implausible height and weight trajectories.^41^, ^42^

The main outcome of incident fracture was determined using prespecified validated lists of International Statistical Classification of Diseases 10th edition (ICD‐10).^43^, ^44^ These fracture codes have been specifically validated within the SIDIAP database before this study, with a positive predictive value of 80.5%.^44^ In this validation study, there was no association found between misclassification of fracture with BMI. Anatomical fracture sites were defined as “axial” (spine/thorax but also including pelvis and clavicle), upper limb (proximal upper limb, wrist/forearm, hand), and lower limb (femur, tibia/fibula/ankle, and foot). In a secondary analysis, anatomical fracture sites were analyzed individually, and this analysis was preplanned.

### Statistical analysis

Cumulative incidence of fracture in childhood (from ages 4 to 15 years) was calculated using actuarial lifetable methods, stratified by fracture site and sex. Kaplan–Meier plots were used to depict cumulative probability of fracture‐free survival stratified by BMI categories.

Finally, proportional hazards Cox regression models were fitted to calculate hazard ratios (HR) and 95% confidence intervals (95% CI) according to BMI categories, adjusted for age at the time of BMI measurement (in months), sex, socioeconomic status (as measured using the ecological deprivation MEDEA index, calculated and categorized into quintiles for those living in an urban environment, in addition to those living in rural areas categorized as “rural”), and nationality (Spanish or other).^45^ The MEDEA index was generated from 2001 census data from five major Spanish regions, with a study undertaken to identify socioeconomic factors associated with standardized mortality ratios. Significant factors (level of education, employment, housing conditions, number of parents in the household) were then used to produce the index. As this index only includes those who live in urban areas, a “rural” category was added into the socioeconomic category for this study to include children who lived in areas outside of the scope of the MEDEA index.

Data cleaning was undertaken in SPSS, data analysis in SPSS and R with graphical results generated in R.^46^, ^47^

## Results

**Figure 1:**
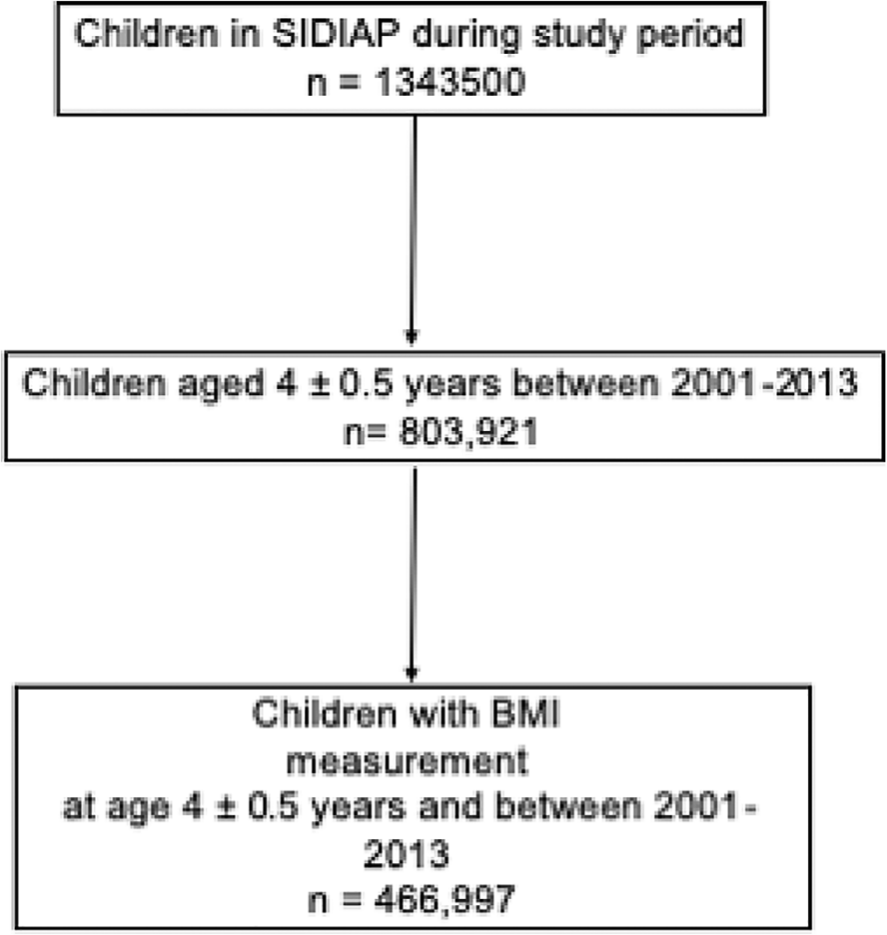
Study participant flow chart.

We identified 466,997 children (out of a total potentially eligible of 803,921) with height and weight measurements taken at a mean age 49.1 months (standard deviation [SD] 2.0) (Fig. 1), and followed them for a median of 4.90 (interquartile range [IQR] 2.50 to 7.61) years. Participants were more commonly of Spanish nationality compared with those excluded (89% versus 81%) but were otherwise similar to those with no 4‐year BMI available (Supplemental Table S1). For those children excluded, the BMI and age given in Supplemental Table S1 are for the first recorded BMI within the data set.

Baseline characteristics of the participants stratified by BMI category are provided in Table 1. Among the participants, 5.7% were considered to have an overweight BMI and 2.0% an obese BMI; there were approximately equal numbers of males and females in the overweight BMI category (female 47.0%) but slightly more boys and more children from more deprived areas in the obese BMI category.

**Table 1:** Baseline Characteristics of the Studied Population

		Total	Underweight range BMI	Normal range BMI	Overweight range BMI	Obese range BMI
Participants	No. (%)	466,997	540 (0.1)	430,681 (92.2)	26,526 (5.7)	9250 (2.0)
BMIz	Mean (SD)	0.46 (1.09)	−3.51 (0.47)	0.8 (0.88)	2.40 (0.28)	3.66 (0.51)
Age in months (at time of BMIz measurement)	Mean (SD)	49.13 (2.00)	49.06 (2.50)	49.13 (1.99)	49.13 (2.01)	49.15 (2.11)
Sex	Female *n* (%)	226,868 (48.6)	248 (45.9)	210,274 (48.8)	12,455 (47.0)	3891 (42.1)
Socioeconomic status (the MEDEA index, quintiles + rural)	1 Least deprived area	57,439 (12.3)	51 (9.4)	53,877 (12.5)	2750 (10.4)	761 (8.2)
	2	68,720 (14.7)	80 (14.8)	63,799 (14.8)	3715 (14.0)	1126 (12.2)
	3	71,416 (15.3)	79 (14.6)	65,660 (15.2)	4170 (15.7)	1507 (16.3)
	4	72,801 (15.6)	79 (14.6)	66,407 (15.4)	4569 (17.2)	1746 (18.9)
	5 Most deprived area	77,129 (16.5)	117 (21.7)	69,581 (16.2)	5259 (19.8)	2172 (23.5)
	Rural *n* (%)	94,825 (20.3)	100 (18.4)	88,571 (20.6)	4666 (17.6)	1488 (1.6)
	Missing	24,667 (5.3)	34 (5.5)	22,786 (5.3)	1397 (5.3)	450 (4.9)
Nationality	Spanish (%)	41,5829 (89.0)	439 (81.3)	383,739 (89.1)	23,568 (88.8)	8083 (87.4)
	Other *n* (%)	51,168 (11.0)	101 (18.7)	46,942 (10.9)	2958 (11.2)	1167 (12.6)

BMI = body mass index; BMIz = body mass index for age *Z*‐score.

**Figure 2:**
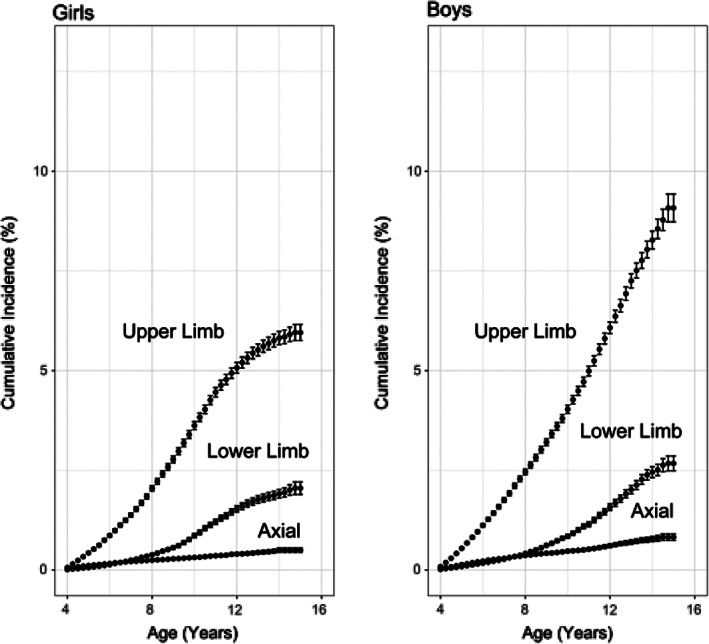
Age‐specific cumulative incidence (95% CI) of fracture stratified by sex and skeletal site affected (upper limb, lower limb, or axial).

Overall, the cumulative incidence of fracture in childhood (from ages 4 to 14 years) was 10.19% (95% CI 9.96% to 10.43%). After stratification by sex, cumulative incidence was 8.24% (95% CI 7.99% to 8.49%) in girls and 12.05% (95% CI 11.66% to 12.44%) in boys. Upper limb fractures were the most commonly affected skeletal sites in both sexes, followed by lower limb, with “axial” fractures being rare in childhood (Fig. 2).

The overall cumulative incidence of fracture during childhood was 9.20% (95% CI 3.79% to 14.61%) for underweight, 10.06% (9.82% to 10.29%) for normal weight, 11.28% (10.22% to 12.35%) for children with an overweight BMI, and 13.05% (10.69% to 15.41%) for children in the obese range BMI category (Supplemental Fig. S1). When analyzed by BMI category and sex, cumulative incidence of fracture during childhood (from ages 4 to 14 years) was higher in children with obesity independent of sex (Supplemental Table S2).

**Figure 3:**
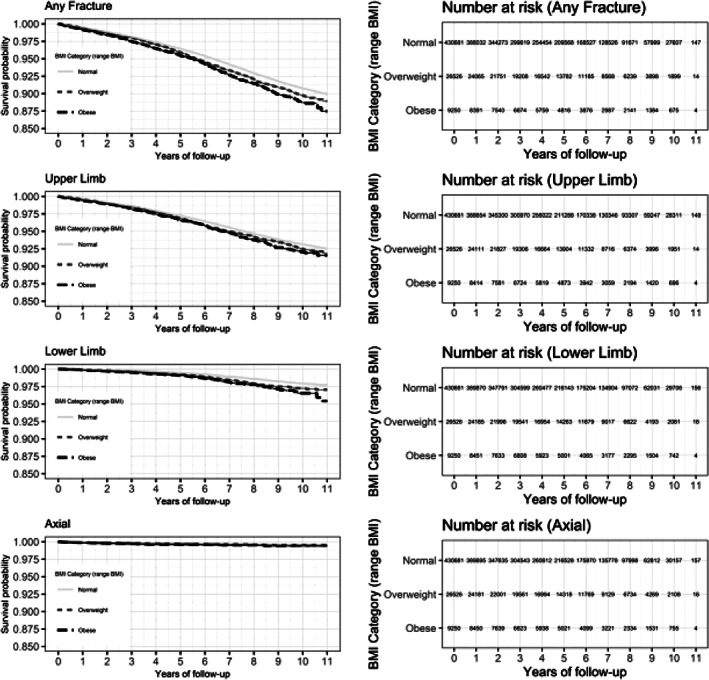
Kaplan–Meier plot for probability of survival (fracture‐free) stratified by BMI category; all fractures.

Cox regression models displayed an association between preschool BMI and childhood fracture. Table 2 shows both unadjusted and adjusted HR for each fracture site according to BMI category, using normal weight as a reference group. The hazard ratios for underweight children are not reported because of small sample size. Overall, the adjusted HR (95% CI) for any fracture was 1.13 (1.07 to 1.20) for children with an overweight BMI and 1.26 (1.15 to 1.37) for children with obesity. The greatest observed association was with lower limb fractures, with adjusted HR = 1.42 (1.26 to 1.59) and 1.74 (1.46 to 2.06) for children in the overweight and obese categories, respectively. A smaller but still significant association was found with upper limb fractures: adjusted HR = 1.10 (1.03 to 1.17) and 1.19 (1.07 to 1.31) for children who were overweight or obese, respectively. Finally, there was no association between increased BMI and axial fracture. Kaplan–Meier plots depicting fracture probability over time stratified by BMI category are shown in Fig. 3. As the proportional hazards assumption was met when tested, no further analysis with BMI as a time‐varying predictor was undertaken.

**Table 2:** Association of Preschool BMI Category and Childhood Fracture

		Unadjusted HR	95% CI	Adjusted^a^ HR	95% CI
Any fracture	Normal range BMI^b^	Ref.		Ref.	
	Overweight range BMI	1.13	1.07 to 1.20	1.12	1.06 to 1.19
	Obese range BMI	1.26	1.15 to 1.37	1.23	1.13 to 1.34
Upper limb fracture	Normal range BMI	Ref.		Ref.	
	Overweight range BMI	1.10	1.03 to 1.17	1.09	1.02 to 1.16
	Obese range BMI	1.19	1.07 to 1.31	1.16	1.04 to 1.29
Lower limb fracture	Normal range BMI	Ref.		Ref.	
	Overweight range BMI	1.42	1.26 to 1.59	1.41	1.26 to 1.58
	Obese range BMI	1.74	1.46 to 2.06	1.72	1.44 to 2.04
Axial fracture	Normal range BMI	Ref.		Ref.	
	Overweight BMI	0.85	0.68 to 1.06	0.85	0.68 to 1.06
	Obese BMI	1.04	0.74 to 1.45	1.02	0.73 to 1.43
					

BMI = body mass index; HR = hazard ratio; CI = confidence interval.

a Adjusted for sex, age (in months), socioeconomic status (the MEDEA index), and nationality.

b Underweight range BMI category not calculated because of small sample size.

In a more granular analysis separating individual fracture sites (Supplemental Table S3), the association between overweight or obese range BMI and fracture risk was more evident for distal fractures, with an almost 40% excess of hand fractures associated with an obese range BMI (adjusted HR = 1.37 [1.14–1.66]), an 80% increase in tibia/fibula fractures (adjusted HR = 1.81 [1.38–2.37]), and an almost 70% increase in foot fractures (adjusted HR = 1.66 [1.32–2.10]). Note that the analyses for thorax/rib cage, spine, and pelvis are not reported here because of insufficient statistical power.

Further analysis was undertaken to determine if there was a differential effect of body mass index for age *Z*‐score (BMIz) upon fracture risk according to nationality. A multiplicative interaction test for nationality and BMIz was run for all models, and the *p* value for interaction was >0.2. Because there was no evidence of a differential effect, no stratification was undertaken.

## Discussion

### Principle findings

This study found an association between elevated preschool BMI and increased fracture incidence in childhood. Having an obese BMI at the time of starting school was associated with a 70% and 20% excess risk of lower and upper limb fractures during childhood. Having an overweight BMI was associated with 40% and 10% excess risk of lower and upper limb fractures. This association of increased fracture incidence was independent of age, socioeconomic status, sex, and nationality. Previous work has suggested higher incidence in boys and urban children, with conflicting evidence of the impact of socioeconomic status.^12^, ^13^, ^14^, ^15^

Secondary analysis in this study showed that associated fracture risk varied depending upon anatomical location, with increased risk associated with distal limb fractures. Previous work in children has also suggested a higher rate of distal upper limb fractures in children with a high BMI compared with other anatomical sites.^24^, ^26^ By comparison, research in adults has found excess risk of distal upper limb fractures associated with an overweight or obese BMI in women (in some studies after adjustment for bone mineral density), and fewer distal fractures in men with increased weight.^8^, ^10^, ^48^, ^49^

### Comparison with other studies

Childhood fracture has traditionally been postulated as a marker of low bone density in those fractures sustained from low‐energy trauma.^17^, ^50^, ^51^, ^52^ Impaired bone strength and lower bone mineral density have been reported in children with fractures, and these pathological changes are superimposed upon the reduced cortical thickness and mineral density that normally accompany early puberty.^52^, ^53^, ^54^

There are several potential causal pathways in which obesity could have a detrimental effect upon childhood bone health. Low levels of vitamin D have been reported in children with obesity, which may be connected to a reduced dietary intake but also due to the proinflammatory state associated with obesity.^23^, ^31^, ^55^, ^56^, ^57^, ^58^ The negative impact of obesity upon bone health could also be explained by altered levels of adipokines and cytokines and reduced osteoblast activity.^29^, ^50^, ^59^, ^60^, ^61^ By contrast, the greater forces going through the limb, reduced physical activity, and impaired balance observed in children with a higher BMI could be the cause of increased fracture incidence.^30^, ^62^, ^63^, ^64^, ^65^, ^66^

The proportion of children with an overweight or obese range BMI in this study is lower than that found in the US (13.9% combined) but similar to worldwide prevalence described by the WHO.^67^, ^68^ The WHO describes that although the largest increase in childhood obesity has been in high‐income countries, there has been an increase in obesity in low‐ and middle‐income countries. High rates of malnutrition continue to exist in sub‐Saharan Africa and Asia, but increasingly obesity coexists alongside malnutrition, with around half of children worldwide who are considered obese or overweight aged 5 years living in Asia.

### Strengths and weaknesses of this study

Our research question is clinically important but difficult and unethical to investigate in a clinical trial setting. Using observational data is therefore necessary, and this study has a unique combination of a population‐based pediatric surveillance program alongside long‐term longitudinal follow‐up and limited migration of children from the region during the study period (only 4.6% migration of the included cohort was observed during the study period). The outcome of fracture incidence is also well defined in the data, with the coding system used in the data set being validated before the study began.^44^

This study used data from a surveillance program for the whole pediatric population in one region where the majority of health care is within the one public system and there was low rates of migration, enabling all children engaging with routine public health care to be included. Demographic analysis confirmed that all socioeconomic groups were represented appropriately in this cohort, and this allows the results to be more generalizable. This cohort is also much larger than those in these previous studies and also included children from both rural and urban communities, reducing the chance of selection bias.

In many observational studies where BMI is used as the representative variable for obesity, there is the risk of introducing selection bias and reverse causality, as BMI is more likely to be recorded in individuals with preexisting health care conditions.^69^, ^70^ We have attempted to minimize the risk of these methodological issues by using data from a universal screening program. We also performed sensitivity analysis, comparing patients with and without a recorded BMI (Supplemental Table S1). Being aware of this risk of selection bias from only including children who engaged with the screening program, we noted the baseline demographics to be similar in those with and without a valid BMI measurement, indicating the cohort is representative of the pediatric population of the region included in SIDIAP.

Our study also has some limitations. First, the proportion of children whose BMI lies within the overweight or obese categories at age 4 years in this region is slightly smaller than in other populations, and therefore external validation in a different population would be beneficial to confirm the generalizability of these results.^1^, ^71^ Second, the use of BMIz as a predictor of obesity in children may have limitations. Although BMI remains the consensus agreed measure for determining obesity, previous work has focused upon validating BMI in slightly older children.^72^, ^73^, ^74^, ^75^ BMIz has been reported as a weak to moderate predictor of total fat mass and percentage body fat in obese and overweight children aged under 9 years when evaluated as part of treatment in a weight management program.^76^, ^77^ In lieu of a more widely used metric of obesity, BMIz was used in this population to make the results more generalizable and comparable to other studies, and we acknowledge that in the future a more precise measurement of adiposity may be used in preschool children.^74^, ^78^

This study has studied the association of BMI recorded at age 4 years with fracture risk during childhood, rather than the trajectory of a child's weight during this period. This preschool period could be considered as the early weight gain occurring in the “adiposity rebound” described to be associated with young adult obesity.^79^, ^80^, ^81^

BMI may underrepresent adiposity in children of Asian descent.^82^ Considering that the non‐Spanish population represents only 11% of the cohort and the majority of the non‐Spanish population in Catalonia are children of Latin American descent, it is less likely to have a significant impact upon results.

Furthermore, although adjustment was undertaken for the many factors implicated in fracture incidence in the literature, the prevalence of participation in sporting activities and general patterns of activity in addition to some medical factors (such as steroid use, chronic kidney disease) within this cohort is unknown. There is some evidence of BMIz being negatively associated with physical activity levels in older children, but elaboration of physical activity levels is not possible within this study of preschool children.^83^

### Future research

This work suggests that interventions to treat obesity in early childhood could have benefits for the primary or secondary prevention of fractures later in childhood, especially in the prevention of fractures within the forearm and hand or foot and ankle. Although initial studies investigating the impact of weight loss upon bone health in children and adolescents have found improved physical activity levels and improved bone mass, further work is needed to determine the overall impact of pediatric weight loss upon bone health, especially considering the evidence for bone density loss associated with weight reduction in adults.^84^, ^85^, ^86^, ^87^, ^88^ This study investigated the impact of BMI at age 4 years to identify those who had an overweight or obese BMI in preschool years, appreciating that this is likely to represent early “adiposity rebound” and those children who are likely to remain overweight during childhood. Future work could also follow the weight trajectory of this population to determine if there is a change in adiposity during childhood that is associated with fracture risk. Similarly, future research could also collect data surrounding lifestyle confounders such as activity levels and enrich with further details of past medical history that could impact upon bone health to further investigate the potential associations found in this study.

## Disclosures

All authors have completed the ICMJE uniform disclosure form at www.icmje.org/coi_disclosure.pdf uploaded with this submission and declare these conflicts of interests that all exist outside of this submitted work: DP‐A reports grants and other from AMGEN; grants, personal fees, and other from UCB Biopharma; grants from Les Laboratoires Servier, outside the submitted work, and Janssen, on behalf of IMI‐funded EHDEN and EMIF consortiums, and Synapse Management Partners have supported training programs organized by DPA's department and open for external participants. CC reports personal fees from Amgen, personal fees from Danone, personal fees from Eli Lilly, personal fees from GSK, personal fees from Kyowa Kirin, personal fees from Medtronic, personal fees from Merck, personal fees from Nestle, personal fees from Novartis, personal fees from Pfizer, personal fees from Roche, personal fees from Servier, personal fees from Shire, personal fees from Takeda, and personal fees from UCB, outside the submitted work. DM‐L reports personal fees and other from AMGEN, personal fees from ITALFARMACO, personal fees from FERRER, personal fees from NOVARTIS, personal fees from Eli Lilly, personal fees from RUBIÓ, outside the submitted work. No other relationships or activities could appear to have influenced the work.

## Supporting information


**Supplemental Table S1.** Baseline characteristics for study participants compared with children excluded due to lack of information on BMI measured at age 4 years ±6 months
**Supplemental Table S2.** Cumulative incidence according to BMIz category and sex
**Supplemental Table S3.** Association between BMIz categories and specific fracture site risks
**Supplemental Fig. S1.** Cumulative Incidence of Fracture According to BMI Category.
