# Supplementary material for: Preschool Obesity Is Associated With an Increased Risk of Childhood Fracture: A Longitudinal Cohort Study of 466,997 Children and Up to 11 Years of Follow‐up in Catalonia, Spain
Source: J Bone Miner Res. 2020 Apr 7;35(6):1022–30. doi: 10.1002/jbmr.3984 (PMC7116071; doi:10.1002/jbmr.3984)

Supplementary Table 1. Baseline characteristics for study participants compared to children excluded due to lack of information on BMI measured at age 4 years ± 6 months old

|  | | | Included patients (BMI available at age 48±6 months) | | | | | Excluded patients (no BMI available at age 48±6 months) | | | |
| --- | --- | --- | --- | --- | --- | --- | --- | --- | --- | --- | --- |
|  | | Total | Underweight range BMI | Normal range BMI | Overweight range BMI | Obese range BMI | Total | Underweight range BMI | Normal range BMI | Overweight range BMI | Obese range BMI |
| Participants | N (%) | 466 997 | 540 (0.1) | 430 681 (92.2) | 26526 (5.7) | 9 250 (2.0) | 336 924 | 2 296 (0.7) | 278 030 (82.5) | 37 052 (11.0) | 19 546 (5.8) |
| BMIz | Mean (SD) | 0.46 (1.09) | -3.51 (0.47) | 0.80 (0.88) | 2.40 (0.28) | 3.66 (0.51) | 0.43 (1.17) | -2.71 (0.64) | 0.11 (0.87) | 1.72 (0.50) | 2.91 (0.73) |
| Age in months at BMIz measurement | Mean (SD) | 49.13 (2.00) | 49.06 (2.50) | 49.13 (1.99) | 49.13 (2.01) | 49.15 (2.11) | 54.30 (36.61) | 85.72 (39.24) | 49.00 (34.58) | 76.69 (36.85) | 83.53 (31.80) |
| Sex | Female N  (%) | 226 868 (48.58) | 248 (45.9) | 210 274 (48.8) | 12 455 (47.0) | 3 891 (42.1) | 162 842 (48.3) | 1 029 (44.8) | 136 061 (48.9) | 17 981 (39.8) | 7 771 (48.5) |
| Socio-economic status (the MEDEA index, quintiles + rural) | 1 Least deprived area | 57 439 (12.3) | 51 (0.1) | 53 877 (93.8) | 2 750 (4.8) | 761 (1.3) | 50 398 (15.0) | 349 (0.7) | 42 208 (83.7) | 5 557 (11.0) | 2 284 (4.5) |
|  | 2 | 68 720 (14.7) | 80 (0.1) | 63 799 (92.8) | 3 715 (5.4) | 1 126 (1.6) | 47 392 (14.1) | 301 (0.6) | 38 886 (82.1) | 5 471 (11.5) | 2 734 (5.8) |
|  | 3 | 71 416 (15.3) | 79 (0.1) | 65 660 (91.9) | 4 170 (5.8) | 1 507 (2.1) | 47 313 (14.0) | 330 (0.7) | 38 274 (80.9) | 5 564 (11.8) | 3 145 (6.6) |
|  | 4 | 72 801 (15.6) | 79 (0.1) | 66 407 (91.2) | 4 569 (6.3) | 1 746 (2.4) | 49 127 (14.6) | 325 (0.7) | 39 808 (81.0) | 5 755 (11.7) | 3 239 (6.6) |
|  | 5 Most deprived area | 77 129 (16.5) | 117 (0.2) | 69 581 (90.2) | 5 259 (6.8) | 2 172 (2.8) | 55 468 (16.5) | 444 (0.8) | 44 928 (81.0) | 6 134 (11.1) | 3 962 (7.1) |
|  | Rural N (%) | 94 825 (20.3) | 100 (0.1) | 88 571 (93.4) | 4 666 (4.9) | 1 488 (1.6) | 59 418 (17.6) | 404 (0.7) | 49 898 (84.0) | 6 110 (10.3) | 3 006 (5.1) |
|  | Missing | 24 667 (5.3) | 34 (0.1) | 22 786 (92.4) | 1 397 (5.7) | 450 (1.8) | 27 808 (8.3) | 143 (0.5) | 24028 (86.4) | 2 461 (8.8) | 1 176 (4.2) |
| Nationality | Spanish N (%) | 415 829 (89.0) | 439 (81.3) | 383 739 (89.1) | 23 568 (88.8) | 8 083 (87.4) | 272 974 (81.0) | 1 557 (67.8) | 225 915 (81.3) | 29 833 (80.5) | 15 669 (80.2) |
|  | Other N (%) | 51168 (10.96) | 101 (18.7) | 46 942 (10.9) | 2 958 (11.2) | 1 167 (12.6) | 63 950 (19.0) | 739 (32.2) | 52 115 (18.7) | 7 219 (19.5) | 3 877 (19.8) |

Supplementary Table 2. Cumulative Incidence according to BMIz category and sex

| Gender | Category | N of fractures^a^ | Cumulative  Incidence (%) | 95% CI | |
| --- | --- | --- | --- | --- | --- |
| Any fracture |  |  |  |  |  |
| Female | Underweight range BMI | <5 |  |  |  |
|  | Normal range BMI | 7932 | 8.13 | 7.87 | 8.40 |
|  | Overweight range BMI | 589 | 9.18 | 8.34 | 10.02 |
|  | Obese range BMI | 212 | 11.01 | 8.81 | 13.21 |
| Male | Underweight range BMI | 14 | 13.84 | 4.66 | 23.02 |
|  | Normal range BMI | 10267 | 11.90 | 11.50 | 12.29 |
|  | Overweight range BMI | 743 | 13.25 | 11.32 | 15.19 |
|  | Obese range BMI | 302 | 14.64 | 10.80 | 18.48 |
| Upper extremity fracture |  |  |  |  |  |
| Female | Underweight range BMI | <5 |  |  |  |
|  | Normal range BMI | 5939 | 5.89 | 5.68 | 6.10 |
|  | Overweight range BMI | 442 | 6.69 | 5.96 | 7.43 |
|  | Obese range BMI | 156 | 7.14 | 5.89 | 8.40 |
| Male | Underweight range BMI | 10 | 10.98 | 2.25 | 19.71 |
|  | Normal range BMI | 7814 | 9.02 | 8.66 | 9.37 |
|  | Overweight range BMI | 539 | 9.84 | 8.01 | 11.67 |
|  | Obese range BMI | 212 | 9.49 | 7.58 | 11.40 |
| Lower extremity fracture |  |  |  |  |  |
| Female | Underweight range BMI | <5 |  |  |  |
|  | Normal range BMI | 1564 | 1.98 | 1.82 | 2.14 |
|  | Overweight range BMI | 142 | 2.59 | 2.12 | 3.05 |
|  | Obese range BMI | 52 | 3.93 | 2.08 | 5.79 |
| Male | Underweight range BMI | <5 | 3.51 | 0.00 | 8.62 |
|  | Normal range BMI | 1806 | 2.54 | 2.37 | 2.71 |
|  | Overweight range BMI | 171 | 3.32 | 2.56 | 4.08 |
|  | Obese range BMI | 82 | 5.92 | 2.45 | 9.39 |
| Axial fracture |  |  |  |  |  |
| Female | Underweight range BMI | <5 |  |  |  |
|  | Normal range BMI | 592 | 0.51 | 0.45 | 0.57 |
|  | Overweight range BMI | 26 | 0.36 | 0.20 | 0.52 |
|  | Obese range BMI | 14 | 0.48 | 0.21 | 0.75 |
| Male | Underweight range BMI | <5 | 1.67 | 0.00 | 3.56 |
|  | Normal range BMI | 917 | 0.85 | 0.76 | 0.93 |
|  | Overweight range BMI | 56 | 0.57 | 0.40 | 0.74 |
|  | Obese range BMI | 21 | 0.64 | 0.32 | 0.96 |

1. *Where numbers less than 5, aggregated to prevent secondary disclosure of data*

Supplementary Table 3. Association between BMIz categories and specific fracture site risks

|  |  | Unadjusted HR | 95.0% CI | Adjusted HR^a^ | 95.0% CI |
| --- | --- | --- | --- | --- | --- |
| Humerus/Proximal arm | Normal range BMI | REF |  | REF |  |
|  | Overweight range BMI | 0.95 | 0.77 to 1.17 | 0.94 | 0.77 to 1.16 |
|  | Obese range BMI | 0.73 | 0.49 to 1.08 | 0.71 | 0.48 to 1.05 |
| Collarbone | Normal range BMI | REF |  | REF |  |
|  | Overweight range BMI | 0.96 | 0.77 to 1.19 | 0.95 | 0.76 to 1.19 |
|  | Obese range BMI | 1.14 | 0.81 to 1.60 | 1.12 | 0.79 to 1.57 |
| Wrist/forearm | Normal range BMI | REF |  | REF |  |
|  | Overweight range BMI | 1.15 | 1.07 to 1.25 | 1.14 | 1.06 to 1.24 |
|  | Obese range BMI | 1.15 | 1.02 to 1.31 | 1.14 | 1.00 to 1.29 |
| Hand | Normal range BMI | REF |  | REF |  |
|  | Overweight range BMI | 1.04 | 0.91 to 1.19 | 1.02 | 0.90 to 1.17 |
|  | Obese range BMI | 1.43 | 1.18 to 1.73 | 1.37 | 1.14 to 1.66 |
| Femur | Normal range BMI | REF |  | REF |  |
|  | Overweight range BMI | 0.71 | 0.33 to 1.52 | 0.70 | 0.33 to 1.48 |
|  | Obese range BMI | 1.75 | 0.77 to 3.95 | 1.63 | 0.72 to 3.69 |
| Tibia / fibula | Normal range BMI | REF |  | REF |  |
|  | Overweight range BMI | 1.57 | 1.31 to 1.87 | 1.57 | 1.31 to 1.87 |
|  | Obese range BMI | 1.81 | 1.38 to 2.37 | 1.81 | 1.38 to 2.37 |
| Foot | Normal range BMI | REF |  | REF |  |
|  | Overweight range BMI | 1.35 | 1.16 to 1.58 | 1.34 | 1.15 to 1.57 |
|  | Obese range BMI | 1.68 | 1.34 to 2.13 | 1.66 | 1.32 to 2.10 |

a) Adjusted for sex, age (in months), socioeconomic status (the MEDEA index) and nationality

Supplementary Figure 1. Cumulative incidence of fracture according to BMI category.


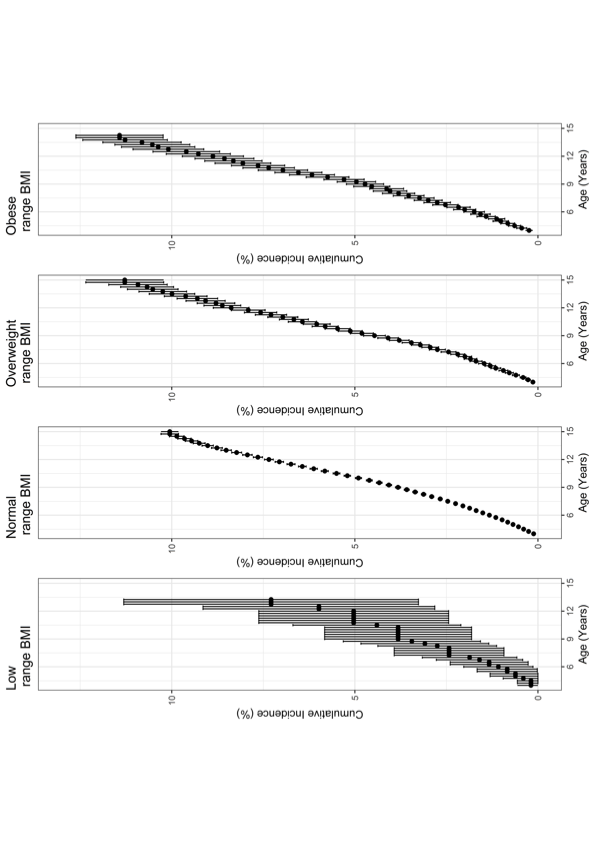

Supplement: Supplementary file 1 — Supplemental Table S1. Baseline characteristics for study participants compared with children excluded due to lack of information on BMI measured at age 4 years ±6 months Supplemental Table S2. Cumulative incidence according to BMIz category and sex Supplemental Table S3. Association between BMIz categories and specific fracture site risks Supplemental Fig. S1. Cumulative Incidence of Fracture According to BMI Category. [file JBMR-35-1022-s001.docx]
